# From opera buffa to opera seria: anniversaries of Royal College of Surgeons of England research initiatives

**DOI:** 10.1093/bjs/znad412

**Published:** 2023-12-14

**Authors:** Peter J Hutchinson, Thomas Pinkney, Midhun Mohan, David Cromwell, Jan van der Meulen, Martyn Coomer, Ralph Tomlinson, Sarah King, Murat Akkulak, Robert Hinchliffe, David J Beard, Dion Morton, Linda Orr, members of the Royal College of Surgeons of England research initiatives, Derek Alderson, Norman Williams, Dion Morton, Martyn Coomer, Michael Rawlins, Richard Ross, Ann Berger, Robert Lechler, Andrew Davies, Dr Kate Law, Arnie Purushotham, Nick Ross, David Cromwell, Ian Lewis, Nicola Keat, Duncan Summerton, Max Parmar, Clare Shaw, Nick Black, Murat Akkulak, Martyn Coomer, Louise Duncan, Nicola Extance-Vaughn, Johnny Fountain, Peter Hutchinson, Sarah King, Dion Morton, Andrew Reed, Linda Slater, Carol Stevenson, Ralph Tomlinson, Scott Willoughby, Jackie Weller, Joy Adamson, David Beard, Michael Douek, Rob Hinchliffe, David Jayne, Michael D. Jenkinson, Cliona Kirwan, Amar Rangan, Tom Pinkney, Jane Blazeby, Julia Brown, Nigel Bundred, Peter Brocklehurst, Marion Campbell, Andy Carr, Julie Croft, Freddie Hamdy, Paula Ghaneh, Iain Hutchison, Pam Kearns, Graeme MacLennan, Laura Magill, Catriona McDaid, Gavin Murphy, James N’Dow, Craig Ramsay, Chris Rogers, Deborah Stocken, David Torgerson, Paul Baker, Matt Bown, Dan Carradice, Filipe Correia-Martins, Peter Friend, Matt Gardiner, Xavier Griffin, Nigel Hall, Douglas Hammond, Michael D. Jenkinson, Robert Jones, Stuart McIntosh, Caroline Moore, Susan Moug, Gavin Murphy, James O’Hara, Daniel Perry, Shelley Potter, Dimitrios Pournaras, Emma Reay, Keith Roberts, George Smith, Tim Underwood, Dale Vimalachandran, Louise Wan, Simon Bach, Jane Blazeby, Matt Costa, Ian Chetter, Adele Francis, Peter Hutchinson, Abhilash Jain, Dae Kim, Jim McCaul, Sam McClinton, Amar Rangan, David Taggart, Anne Schilder, Richard Kerr, Angelos Kolias, Nuha Yassin, Rachel Hargest, Rocco Friebel, Peter Morris, Peter Bell, Anthony Mundy, Norman Williams, Derek Alderson, Neil Mortensen, Tim Rockall, Cliff Shearman, Peter Friend, Ian Loftus, Norman Browse, Rodney Sweetnam, Barry Jackson, Peter Morris, Hugh Phillips, Lord Bernard Ribeiro, John Black, Norman Stanley Williams, Dame Clare Marx, Derek Alderson, Neil Mortensen, Tim Mitchell

**Affiliations:** The Royal College of Surgeons of England, London, UK; The Royal College of Surgeons of England, London, UK; The Royal College of Surgeons of England, London, UK; The Royal College of Surgeons of England, London, UK; The Royal College of Surgeons of England, London, UK; The Royal College of Surgeons of England, London, UK; The Royal College of Surgeons of England, London, UK; The Royal College of Surgeons of England, London, UK; The Royal College of Surgeons of England, London, UK; The Royal College of Surgeons of England, London, UK; The Royal College of Surgeons of England, London, UK; The Royal College of Surgeons of England, London, UK; The Royal College of Surgeons of England, London, UK

## Introduction

This year, 2023, is the anniversary of three initiatives of the Royal College of Surgeons of England: 30 years of the Research Fellowship Scheme, 25 years of the Clinical Effectiveness Unit, and 10 years of the Clinical Research Initiative. During the previous century it was argued that surgical clinical research was faltering, and 27 years have elapsed since Richard Horton’s now infamous editorial ‘Surgical research or comic opera: questions, but few answers’ was critical of surgical research^1^. The editorial went on to state ‘cynics might even claim the personal attributes that go to make a successful surgeon differ from those needed for collaborative multicentre research’. Furthermore, Horton quoted the words of medical statistician Major Greenwood from 1923, ‘I should like to shame (surgeons) out of the comic opera performances which they suppose are statistics of operations’. Horton stated that, in surgery journals, only 7% of papers reported data from RCTs and case series were the most common type of manuscript. Horton concluded that logic insists that a large proportion of the surgical literature is of questionable value and proposed that only when the quality of publications in the surgical literature has improved will surgeons reasonably be able to rebut the charge that as much as half of the research they undertake is misconceived.

Richard Horton was certainly harsh, but were Horton’s statements fair? Perhaps an analysis of the literature would have shown that the same criticisms were applicable to other specialties. However, there were issues in terms of surgical research. It could be argued that, historically, surgeons focused on practical surgical competence, training, and management rather than research, and funding agencies focused on basic science and translational research. Spending on surgical research was less than 2% of the total research budget, yet 30% of National Health Service (NHS) patients received surgical care.

However, 30 years ago, a number of research initiatives were introduced, which created change and has resulted in a substantial shift in surgical research culture^2^. The Royal College of Surgeons of England Research Fellowship Scheme commenced in 1993 and has trained a generation of surgeons with an interest in basic, translational, and clinical research. The Clinical Effectiveness Unit founded in 1998 has developed audit and research methodologies and has produced evidence on clinical effectiveness and cost-effectiveness. After the turn of the century, there has been an exponential growth in clinical research, including trials (specifically RCTs). The infrastructure underpinning this evolution has been three-fold. First, the birth of the National Institute for Health Research (now called the National Institute for Health and Care Research (NIHR)). The NIHR has played a fundamental role both in terms of access to funding and delivery of research via the Clinical Research Networks and through NIHR infrastructure within NHS trusts supporting the delivery of clinical research. Second, the formation of the Royal College of Surgeons of England Clinical Research Initiative, and, third, a growth in methods suitable for the design and delivery of surgical trials, much of which was funded by investment into the Medical Research Council (MRC) 2010 initiative ‘Hubs for Trials Methodology Research’. More recently, the Research Excellence Framework has accelerated the change, and created a greater emphasis towards impact, which the surgical research environment is well placed to deliver.

A new surgical culture that places emphasis on conducting high-quality research to advance patient care is now firmly established in the UK. In turn, this has led to a growth in surgical research output. There is now a substantial cohort of surgeons determined to deliver high-quality research; importantly, this includes, not just academic consultants, but also NHS consultants, who form the majority of the surgical workforce in the UK. The role of trainees, particularly the major impact of trainee collaboratives, cannot be overstated. Moreover, collaboration with other specialties and methodologists has been crucial. Horton stated in the *Lancet* editorial that ‘to retain their academic reputation surgeons must find imaginative ways to collaborate with epidemiologists to improve the design of the case series and to plan randomised trials’; trial methodologists, statisticians, health economists, database programmers, qualitative researchers, research nurses, and patient representatives can now be added as integral members of surgical research. The role of the Clinical Trials Units and the NIHR Clinical Research Networks infrastructure have also been critical in the successful delivery of surgical research. This is reflected in the quality of surgical research output beyond the UK and across the world. See Box 1.

Box 1 **Lessons learnt: how to set up a surgical research initiative**

**Table:** 

Work with patients to define important surgical research questions Establish a research committee within the appropriate national surgical college Engage with funders: government funding streams, philanthropists, charitable trusts, and foundations Define a research strategy focusing on individuals (fellowships) and projects (e.g. specific trial funding), and the creation of a dynamic and collaborative community of researchers Support trainees to establish research collaboratives Utilize a model of collaborative authorship Focus on impact (using results to define guidelines and change practice)

## Royal College of Surgeons of England Research Fellowship Scheme

The Royal College of Surgeons of England Research Fellowship Scheme gives trainees, and now also specialty and specialist doctors (SAS) surgeons, an opportunity to dedicate an interval of time solely to research. Founded in 1993, following a case to Council from then President Sir Norman Browse, with Sir Peter Morris as its first Chair, the aim of the scheme has been to facilitate new knowledge acquisition that will translate over time into improved surgical outcomes. In addition, and perhaps as importantly, it aims to encourage more surgical trainees to view research as an important part of their training. The scheme is designed to identify and encourage those who wish to pursue an academic surgical career with a major ongoing commitment to research, but also to support those future NHS surgeons who hope to deliver a research portfolio outside a formal academic appointment.

During the past 30 years (with the support of many highly generous and often long-standing funders) over 900 1-year fellowships have been awarded, amounting to funding in the region of €58 million. Approximately 15–25 awards are made annually and an average award covers total salary costs at the level of an ST4–ST8 surgical trainee, consumables up to €3500, and a travel award, and incorporates a research methods course run by the Clinical Effectiveness Unit. The fellowship scheme has had a dramatic impact. Within the first 15 years, 91% of fellowship recipients had successfully obtained a higher degree and 60% had obtained funding for future research with over half of this cohort receiving grants from national research funding bodies^3^.

An impact analysis spanning the whole 30 years is underway and expected to be even more encouraging. Numerous examples of basic scientific discovery led by fellowship recipients have now translated into patient benefit in the UK and beyond, with many having a truly global reach. The scope of topics investigated is vast and ranges from pure cellular biology, trauma systems, surgical diagnostics, robotics, and other devices through to ‘how green are our theatres?’ and, critically, ‘how do we make them better?’. National and international collaborations within and beyond the recognized ‘medical research world’ have been made and endure. There are now several generations of consultant surgeons in a position, not only to continue delivery of groundbreaking research, but to inspire the next generations to understand and value the critical importance of surgical research enquiry. This has never been better shown than in the *BMJ*’s 2019 ‘role model’ column, with the first female Professor of Urology (and former Royal College of Surgeons of England Research Fellow) Caroline Moore being nominated by Professor Mark Emberton—himself a former researcher within the Royal College of Surgeons of England Clinical Effectiveness Unit^4^.

## Royal College of Surgeons of England Clinical Effectiveness Unit

The Clinical Effectiveness Unit was established in March 1998 as an academic collaboration between the Royal College of Surgeons of England and the London School of Hygiene and Tropical Medicine (LSHTM). It has had three Directors during its 25 years: Barnaby Reeves (1998–2002), Jan van der Meulen (2002–2011), and David Cromwell (2011–to date). Its original objectives were to carry out national surgical audits and to develop better audit methods, as well as to produce evidence on clinical effectiveness and cost-effectiveness. Early clinical audits were established in collaboration with various medical associations and included studies on outcomes after total hip replacement, sinonasal surgery, and tonsillectomy^5^. Key research studies included a systematic review of thoracic surgical techniques^6^ and a RCT of self-management for men with lower urinary tract symptoms^7^.

Between 2005 and 2013, the Clinical Effectiveness Unit expanded its work regarding national clinical audits, partly reflecting the National Clinical Audit and Patient Outcomes Programme (NCAPOP) established for NHS services in England and Wales. The Clinical Effectiveness Unit again worked with various medical/healthcare organizations to design and deliver a range of national studies, including audits of mastectomy and breast reconstruction, oesophagogastric cancer, the CRANE register of cleft lip and palate abnormalities, and liver/cardiac transplantation^8^. From 2013, the Clinical Effectiveness Unit expanded its activity, delivering national audits of vascular surgery and four cancers (bowel cancer, prostate cancer, oesophagogastric cancer, and breast cancer)^9^. Three of these audits (bowel cancer, oesophagogastric cancer, and vascular surgery) also supported the government’s surgical outcomes initiative, which saw the publication of surgeon-level outcomes for NHS England^10^. This initiative was controversial, but an evaluation by the Clinical Effectiveness Unit suggested that it had a positive impact for patients who had elective surgery for bowel cancer^11^. In 2022, the Clinical Effectiveness Unit was awarded the National Cancer Audit Collaborating Centre, which will deliver all 10 national cancer audits for England and Wales^12^.

An essential part of the Clinical Effectiveness Unit strategy is to consider projects as epidemiological studies of the quality of clinical care. Many national clinical audits can be viewed as multicentre, population-based, cohort studies, and key elements in their study designs correspond to important components of observational studies. Consequently, the generation of high-quality evidence on the processes and outcomes of care requires: a representative sample of patients; well-defined measures of clinical processes and patient outcomes and their determinants; and appropriate methods of analysis.

The Clinical Effectiveness Unit continuously seeks to develop new and better methods. Early on, the potential of linking clinical audit data sets to national administrative databases was recognized, notably the Hospital Episode Statistics (‘HES’) database that captured all admissions in NHS England hospitals. This enabled audits to generate more information about clinical outcomes, such as long-term revision rates for the National Joint Registry^13^, as well as information on patient characteristics, such as co-morbidity burden and frailty^14,15^, which has been important in deriving risk-adjusted indicators to benchmark the performance of healthcare providers.

Another innovation was to complement clinical measures with patient-reported outcome measures (PROMS). The Clinical Effectiveness Unit first used PROMS in the sinonasal surgery audit and the mastectomy and breast reconstruction audit^16,17^. Further developmental work saw them adopted by NHS England in its programme to measure outcomes after elective surgery. PROMS were also used in an evaluation of surgical outcomes from independent sector and NHS hospitals^18^.

A final feature of Clinical Effectiveness Unit projects has been multidisciplinary project teams, which typically have two to three clinical leads working alongside Clinical Effectiveness Unit staff with expertise in health services research, statistics, project management, and clinical epidemiology. Whenever possible, a clinical fellow is part of the team. These surgical and medical trainees take 2–3 years out of training to work on the project and typically enrol in a higher degree. To date, 12 clinical fellows have been awarded an MD or PhD.

## Royal College of Surgeons of England Clinical Research Initiative

The Royal College of Surgeons of England Clinical Research Initiative was established in 2013. It was the vision of Presidents Professor Sir Norman Williams and Professor Derek Alderson, and the first Director of Clinical Research, Professor Dion Morton. The initiative, initially made possible thanks to generous support from Rosetrees Trust comprised three components: the Surgical Trials Centres; the Surgical Specialty Leads (SSLs) and trainee Associate SSLs; and the Surgical Research Collaborative Networks. In 2018, the initiative was further expanded with the appointment of dedicated Chairs in Surgical Trials.

The initiative works closely with the NIHR both in terms of funding (for example via the NIHR Health Technology Assessment (HTA) Prioritization Committee) and delivery of research via the NIHR Clinical Research Networks. The number of open NIHR portfolio surgery-led trials has increased from 34 in 2011 to 188 in 2023. In collaboration with the NIHR, there have been over 601 000 patients entered into trials led or co-managed by surgery, across 1317 studies in the NIHR Clinical Research Networks portfolio, since 2011.

There are now 25 SSLs, nine Chairs, and nine Surgical Trials Centres. The SSLs lead their own trials, but the ethos of the SSL role is to develop new Chief Investigators across the NHS. The Chairs have expertise in both the design and delivery of trials. The Surgical Trials Centres are recognized for their expertise in surgical trial methodology and offer a portal/conduit into trial delivery and conduct. See Boxes 2 and 3.

Box 2 **Trial example: Patient/population–Intervention–Comparison–Outcomes (PICO) (abdominal surgery)**

**Table:** 

**Research question:** To determine the clinical effectiveness of wound edge protection devices in reducing surgical site infection after abdominal surgery. **Design:** Multicentre observer blinded RCT. **Patient/population:** Patients undergoing laparotomy at 21 UK hospitals. **Intervention:** Wound edge protection device during surgery. **Comparison:** Standard care. **Outcomes:** A total of 760 patients were enrolled, with 382 patients assigned to the device group and 378 patients assigned to the control group. Six patients in the device group and five patients in the control group did not undergo laparotomy. Fourteen patients, seven in each group, were lost to follow-up. A total of 184 patients experienced surgical site infection within 30 days of surgery, 91 of 369 (24.7%) in the device group and 93 of 366 (25.4%) in the control group (OR 0.97, 95% c.i. 0.69 to 1.36; *P* = 0.85). **Impact:** Wound edge protection devices do not reduce the rate of surgical site infection in patients undergoing laparotomy, and therefore their routine use for this role cannot be recommended. Pinkney TD, Calvert M, Bartlett DC, Gheorghe A, Redman V, Dowswell G *et al*. Impact of wound edge protection devices on surgical site infection after laparotomy: multicentre randomised controlled trial (ROSSINI Trial). *BMJ* 2013;**347**:f4305

Box 3 **Trial example: Patient/population–Intervention–Comparison–Outcomes (PICO) (orthopaedic surgery)**

**Table:** 

**Research question:** To determine the best management strategy between reconstructive surgery and non-surgical treatment for patients with a non-acute anterior cruciate ligament injury and persistent symptoms of knee instability. **Design:** Multicentre RCT. **Patient/population:** Patients with non-acute anterior cruciate ligament knee instability at 29 UK hospitals. **Intervention:** Surgery (reconstruction). **Comparison:** Physiotherapy (rehabilitation). **Outcomes:** A total of 316 patients were enrolled, with 156 patients assigned to the surgical reconstruction group and 160 patients assigned to the rehabilitation group. Mean(s.d.) knee injury and osteoarthritis outcome score at 18 months was 73.0(18.3) in the surgical group and 64.6(21.6) in the rehabilitation group. The adjusted mean difference was 7.9 (95% c.i. 2.5 to 13.2; *P* = 0.0053) in favour of surgical management. A total of 65 (41%) of 160 patients allocated to rehabilitation underwent subsequent surgery according to protocol within 18 months. **Impact:** Surgical reconstruction as a management strategy for patients with non-acute anterior cruciate ligament injury with persistent symptoms of instability was clinically superior and more cost-effective in comparison with rehabilitation management. Beard DJ, Davies L, Cook JA, Stokes J, Leal J, Fletcher H *et al*. Rehabilitation versus surgical reconstruction for non-acute anterior cruciate ligament injury (ACL SNNAP): a pragmatic randomised controlled trial. *Lancet* 2022;**400**:605–615

Trainee engagement has been fundamental both at an individual level and in terms of the trainee research collaboratives. The SSLs work with Associate SSLs in developing and delivering research in individual specialties. The trainee research collaboratives have been instrumental in delivering both their own research studies and contributing to recruitment. Over 15 000 doctors and medical students from 155 countries have entered patients into collaborative studies and over 150 000 patients have now been entered into collaborative audits, cohort studies, or RCTs, with over 100 publications directly citing collaborative research methodology. The trainee collaborative concept pioneered in surgery has also been developed by other specialties, including geriatric medicine, trauma, anaesthetics, gastroenterology, haematology, psychiatry, and general practice. The same concept has also been developed by medical students, embodied in Student Audit and Research in Surgery (STARSurg), who met in Edinburgh this year, with virtual attendance across three continents. The contribution of trainees to clinical trials is now being formally recognized by two mechanisms: first, through the collaborative authorship model, so that contributors are appropriately indexed in PubMed; and second, through the Royal College of Surgeons of England-NIHR Associate Principal Investigator Scheme. The status of Associate Principal Investigator is awarded to the trainee Principal Investigator for each trial/study site. Empowering students and trainees to lead and partake in high-impact collaborative studies is fundamental to ensure a culture of research. Furthermore, advocating collaboration and collaborative authorship transforms single-centre, small-volume studies into higher-impact, national, multicentre studies. The act of collaboration between students and trainees via surgical research has further instilled a culture of networking, sharing ideas, and cross-platform learning, not seen before in medical education.

## Other Royal College of Surgeons of England research initiatives

Other research initiatives include Royal College of Surgeons of England collaborations between the NIHR Global Surgery Unit (Birmingham) and NIHR Research Groups on Global Surgical Technologies (Leeds), Neurotrauma and Acquired Brain and Spine Injury (Cambridge), Physical Trauma from Injury & POsT Conflict (iPrOTeCT) (Imperial), Burns (Swansea), Equitable Access to Quality Health Care for Injured People (Equi-injury) (Birmingham), and Perioperative and Critical Care (Barts). The Royal College of Surgeons of England also collaborates with the London School of Economics and Political Science through the Global Surgery Policy Unit.

The early initiatives have also been successful at creating key global links to further the development of surgical trials worldwide, such as in Australia (Royal Australasian College of Surgeons), and now with a major emphasis on surgical research in low- and middle-income countries.

The Royal College of Surgeons of England ran a Covid Research Group^19^ with over 50 projects looking at the impact of COVID-19 on surgical patients and surgical services, which has now evolved into a research recovery group. Research into robotics is coordinated through a dedicated Royal College of Surgeons of England Robotics Group (RADAR).

## Future plans and initiatives

Future plans for Royal College of Surgeons of England research include further awards of research fellowships, expansion of the trials initiative, including new trial designs (for example platform trials), growing global surgery, green surgery research, and working with NHS England on the Outcomes and Registries Programme and the Medical Device Outcome Registry, which will include the concept of registry-embedded trials. Working with the Learning Department is providing opportunities for new modules and courses (funded by NIHR Learn). Links are being developed with scientists at the Crick through a buddying scheme; the concept of a surgeon in the laboratory and a scientist in the operating theatre will improve the understanding of the relationship between the pathophysiology of surgical diseases and the way they are treated.

Finally, there is increasing awareness of the importance of impact with a recognition that it is necessary to go beyond delivery of surgical research and generation of evidence to change practice and policy to deliver quality improvement. Current and future initiatives aim to address this through quality-improvement collaboratives, implementation science, and metrics illustrated through, for example, infographics. Registries, as well as assisting in the delivery of trials, can be used to illustrate changes in practice. Other examples of impact include adoption by the National Institute for Health and Care Excellence (‘NICE’) and other guidelines, as well as updates to Good Surgical Practice and other Royal College of Surgeons of England Good Practice Guides.

## Conclusion

Over the past 30 years there has been a marked change in the quality and quantity of UK surgical research. This has been achieved through improved leadership and infrastructure, engaging with government, philanthropists, charitable trusts, and foundations to secure funding streams, supporting trainees, utilizing the model of collaborative authorship, and working with patients to define important research questions. The next stage of delivering high-quality UK research will include continuing to support trainees and research fellowships, novel efficient trial design, increased utilization of registries, and the growth of international collaboration.

## Collaborators

**Members of the Royal College of Surgeons of England research initiatives**

***Instrumental figures in setting up and supporting the initiatives from the onset***

Professor Derek Alderson; Professor Sir Norman Williams; Professor Dion Morton; Martyn Coomer; Professor Sir Michael Rawlins; Richard Ross of Rosetrees Trust; Ann Berger of Rosetrees Trust.

***Members of the Clinical Research Initiative Steering Committee (past and present)***

Professor Sir Robert Lechler; Andrew Davies; Dr Kate Law; Professor Arnie Purushotham; Nick Ross; Professor David Cromwell; Ian Lewis; Nicola Keat; Professor Duncan Summerton; Professor Max Parmar; Dr Clare Shaw; Professor Sir Nick Black.

***Royal College of Surgeons of England staff (past and present)***

Murat Akkulak; Martyn Coomer; Louise Duncan; Nicola Extance-Vaughn; Johnny Fountain; Peter Hutchinson; Sarah King; Professor Dion Morton; Andrew Reed; Linda Slater; Carol Stevenson; Ralph Tomlinson; Scott Willoughby; Jackie Weller.

***Royal College of Surgeons of England Chairs***

Professor Joy Adamson; Professor David Beard; Professor Michael Douek; Professor Rob Hinchliffe; Professor David Jayne; Professor Michael D. Jenkinson; Professor Cliona Kirwan; Professor Amar Rangan; Professor Tom Pinkney.

***Royal College of Surgeons of England Surgical Trials Centre Directors (past and present)***

Professor Jane Blazeby; Professor Julia Brown; Professor Nigel Bundred; Professor Peter Brocklehurst; Professor Marion Campbell; Professor Andy Carr; Julie Croft; Professor Freddie Hamdy; Professor Paula Ghaneh; Professor Iain Hutchison; Professor Pam Kearns; Professor Graeme MacLennan; Dr Laura Magill; Professor Catriona McDaid; Professor Gavin Murphy; Professor James N’Dow; Professor Craig Ramsay; Professor Chris Rogers; Professor Deborah Stocken; Professor David Torgerson.

***Current Royal College of Surgeons of England Surgical Specialty Leads***

Professor Paul Baker; Professor Matt Bown; Mr Dan Carradice; Mr Filipe Correia-Martins; Professor Peter Friend; Mr Matt Gardiner; Professor Xavier Griffin; Mr Nigel Hall; Mr Douglas Hammond; Michael D. Jenkinson; Mr Robert Jones; Mr Stuart McIntosh; Professor Caroline Moore; Professor Susan Moug; Professor Gavin Murphy; Mr James O’Hara; Professor Daniel Perry; Ms Shelley Potter; Mr Dimitrios Pournaras; Ms Emma Reay; Professor Keith Roberts; Mr George Smith; Professor Tim Underwood; Mr Dale Vimalachandran; Ms Louise Wan.

***Past Royal College of Surgeons of England Surgical Specialty Leads***

Mr Simon Bach; Professor Jane Blazeby; Professor Matt Costa; Professor Ian Chetter; Professor Adele Francis; Professor Peter Hutchinson; Professor Abhilash Jain; Professor Dae Kim; Professor Jim McCaul; Mr Sam McClinton; Professor Amar Rangan; Professor David Taggart; Professor Anne Schilder.

***Other Contributors***

Mr Richard Kerr (the future of surgery) Mr Angelos Kolias (trainee collaboratives); Ms Nuha Yassin (fellowship scheme).

***Global Surgery Policy Unit***

Rachel Hargest; Rocco Friebel.

***Chairs of the Royal College of Surgeons of England Research Committee***

Professor Sir Peter Morris; Professor Sir Peter Bell; Professor Anthony Mundy; Professor Sir Norman Williams; Professor Derek Alderson; Professor Neil Mortensen; Professor Tim Rockall; Professor Cliff Shearman; Professor Peter Friend; Professor Ian Loftus.

***Presidents of the Royal College of Surgeons of England***

Sir Norman Browse; Sir Rodney Sweetnam; Sir Barry Jackson; Sir Peter Morris; Hugh Phillips; Lord Bernard Ribeiro; John Black; Sir Norman Stanley Williams; Dame Clare Marx; Professor Derek Alderson; Professor Neil Mortensen; Tim Mitchell.

## Data Availability

Not applicable.
